# Supplementary material for: The role of the interactome in the maintenance of deleterious variability in human populations
Source: Mol Syst Biol. 2014 Sep 26;10(9):752. doi: 10.15252/msb.20145222 (PMC4299661; doi:10.15252/msb.20145222)
Supplement: Supplementary file 1 — Supplementary Figure S1 [file msb0010-0752-SD1.docx]

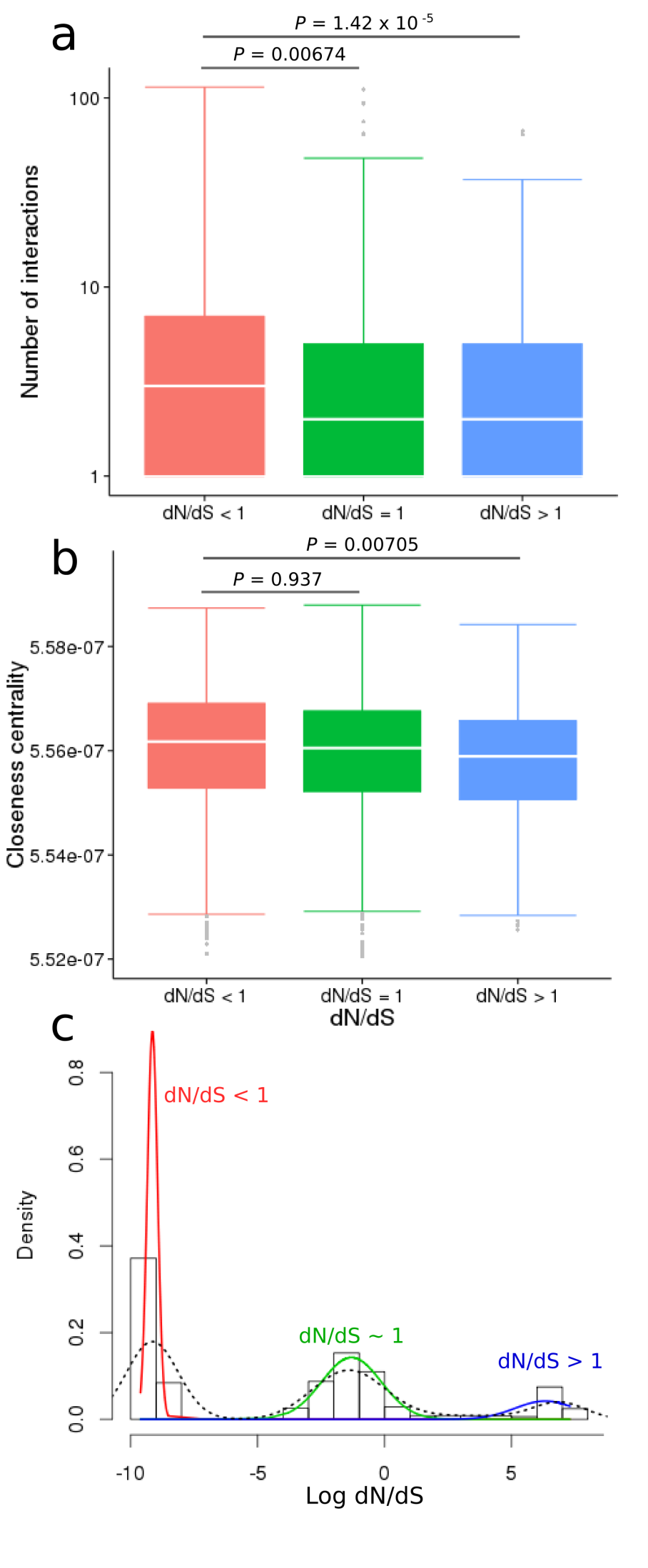


**Supplementary Figure S1**. Boxplots displaying the trends which proteins under positive selection (with a ratio of non-synonymous (dN) to synonymous (dS)mutations greater than 1), neutral selection (with dN/dS approximately equal to 1) and negative selection (dN/dS<1), obtained as in ([Serra et al, 2011](#_ENREF_78)), with respect to different network properties: A) Number of interactions and B) Closeness centrality. The significance level of the mean differences is given as the Mann Whitney U rank sum P value. C) Density plots for the Log dN/dS values using the *normalmixEM* procedure, from the R "mixtools" package, with default parameters.
