# Supplementary material for: The role of the interactome in the maintenance of deleterious variability in human populations
Source: Mol Syst Biol. 2014 Sep 26;10(9):752. doi: 10.15252/msb.20145222 (PMC4299661; doi:10.15252/msb.20145222)
Supplement: Supplementary file 2 — Supplementary Figure S2 [file msb0010-0752-SD2.docx]

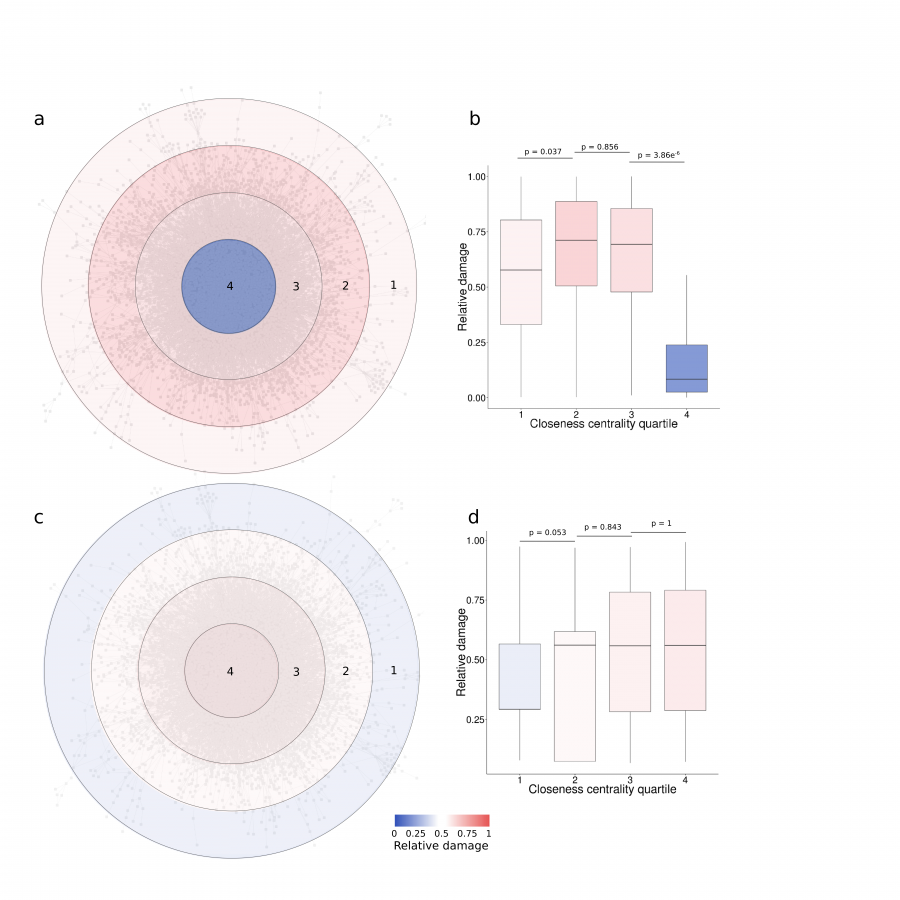


**Supplementary Figure S2. Distribution of proteins carrying deleterious variants across the interactome in normal populations and in somatic of CLL patients.** The relative damage value is the observed frequency of proteins carrying deleterious variants with respect to the expected value obtained from the simulation. This value is taken as an indication of over or under-abundance of proteins with deleterious mutations. The interactome was divided into four sectors containing the quartiles of proteins according to their closeness centrality (from 1, peripheral to 4, central). The color code represents the relative damage value. Such values range between 0 (no proteins affected at all in this quartile) to 1 (the maximum possible number of proteins affected in this quartile). Blue indicates that the frequency of damaging variants is below the median in the simulated individuals (which would correspond to a value of 0.5) whereas red indicates that the value is above the median. A) Distribution of proteins with deleterious variants in the 1078 individuals from the 1000 Genomes populations plus the 252 individuals of the MPG1 Spanish population and the 41 germinal CLL exomes across the interactome. B) The corresponding boxplots representing the distribution of relative damage in each individual in any of the quartiles. There is a significant trend of accumulation of mutations as the quartiles are more peripheral. C) Distribution of proteins with deleterious variants in the 41 somatic CLL exomes, representative of a pathological condition, across the interactome. D) Boxplots representing the distribution of relative damage of each individual in any of the quartiles for the 41 somatic CLL exomes. Opposite to the above, there is a trend of accumulation of mutations in more central quartiles.
